# Factors Associated with Depressive Symptoms in Young Adults with Coronary Artery Disease: Tehran Heart Center's Premature Coronary Atherosclerosis Cohort (THC-PAC) Study

**Published:** 2016-10

**Authors:** Seyed Hesameddin Abbasi, Seyed Ebrahim Kassaian, Saeed Sadeghian, Abbasali Karimi, Soheil Saadat, Flora Peyvandi, Arash Jalali, Tahereh Davarpasand, Shahin Akhondzadh, Nazila Shahmansouri, Masoumeh Lotfi-Tokaldany, Maryam Amiri Abchouyeh, Farah Ayatollahzade Isfahani, Frits Rosendaal

**Affiliations:** 1 Tehran Heart Center, Tehran University of Medical Sciences, Tehran, Iran.; 2 Sina Trauma Research Center, Tehran University of Medical Sciences, Tehran, Iran.; 3 Fondazione IRCCS Ca’ Granda Ospedale Maggiore Policlinico Milano, Angelo Bianchi Bonomi Hemophilia and Thrombosis Centre, Milan, Italy.; 4 Department of Pathophysiology and Transplantation, Università degli Studi di Milano, Milan, Italy.; 5 Psychiatric Research Center, Roozbeh Psychiatric Hospital, Tehran University of Medical Sciences, Tehran, Iran.; 6 Department of Clinical Epidemiology and Department of Thrombosis and Haemostasis, Leiden University Medical Center, Leiden, the Netherlands.

**Keywords:** *Association*, *Cohort Studies*, *Coronary Artery Disease*, *Depression*, *Young Adult*

## Abstract

**Objective:** Depressed coronary artery disease (CAD) patients may experience a poorer prognosis than non-depressed patients. The aim of this study was to find the associated factors for depressive symptoms in young adults with CAD.

**Method:** This was a cross-sectional study within Tehran Heart Center's Premature Coronary Atherosclerosis Cohort (THC-PAC) study. Young adult CAD patients (men ≤ 45 year-old and women ≤ 55 year-old) were visited from March 2013 to February 2014. Demographic, clinical and laboratory data were collected and all patients were asked to fill in the Beck Depression Inventory II. Informed consent was obtained from all participants. A logistic regression model was used to find multiple associated factors of depressive symptoms.

**Results:** Seven hundred seventy patients (mean ±SD age: 45.34 ±5.75 y, men: 47.7%) were visited. The point prevalence of depressive symptoms was 46.9% in women and 30.2% in men (p < 0.001). Logistic regressions model revealed that the most important associated factors for depressive symptoms in the male premature CAD patients were opium usage (OR: 2.4, 95% CI: 1.33-4.43), major adverse cardiac events (MACE) (OR: 2.2, 95% CI: 1.17-3.93), initial coronary artery bypass grafting (CABG) treatment (OR: 2.1, 95% CI: 1.07-4.06), positive family history for CAD (OR: 1.8, 95% CI: 1.11-3.01) and cigarette smoking (OR: 1.7, 95% CI: 0.97-2.98). Hypertension showed a protective role in this group of patients (OR = 0.5, CI = 0.29-0.92). In the female patients, hypertension (OR = 1.5, CI = 0.96-2.22) and body mass index (BMI) (OR = 1.1, CI = 1.02-1.10) were associated with depressive symptoms.

**Conclusion**: In premature CAD male patients, opium usage, MACE, initial CABG treatment, positive family history for CAD and cigarette smoking were associated with depressive symptoms; and hypertension and BMI were associated with depressive symptoms in women.

Coronary artery disease (CAD) is the leading cause of death in many parts of the world. CAD in young adults, referred to as premature CAD, is a challenging medical condition, particularly in the Middle East (1), 

and prognosis of CAD seems to be poorer in young patients than in the elders (2). Meanwhile, depression has been implicated in both the etiology and progression of CAD (3). Depression is one the most prevalent global psychiatric disorders and it has been estimated that point prevalence of depression in the general population varies from 4 to 7% (4). In CAD patients, the point prevalence of depression is higher and has been reported to be from 14 to 47% (3). There is a two-way relationship between depression and CAD. While patients with CAD are more likely to develop depression, depressed individuals have up to two times higher risk of developing CAD than non-depressed persons (5, 6). Furthermore, it has been reported that after controlling for risk factors and disease severity, risk of mortality due to cardiac disease is fourfold higher in patients with major depression than in those without depression (7). 

Since depression is a particular predictor of CAD progression (3), it is crucial to find those CAD patients who are depressed or are at increased risk of depression. Early detection of these high- risk patients may help their cardiologists to reduce CAD progression by appropriate psychiatric interventions. It is not well understood which CAD patient is most susceptible to depression. This study, a substudy of Tehran Heart Center's Premature Coronary Atherosclerosis Cohort (THC-PAC) study, was designed to identify the putative associated factors with depressive symptoms in premature CAD patients (8).

## Materials and Method

***Design and Participants***

Tehran Heart Center's Premature Coronary Atherosclerosis Cohort (THC-PAC) study aimed to examine the progression of CAD in young adults. The methods of this study have been described elsewhere (8).

THC-PAC study comprises a cohort of CAD patients, males ≤ 45 years old and females ≤ 55 years old, with pretty the same sex distribution. Unfortunately, there is no precise and globally accepted definition for premature patients. However, most of the previously done studies have considered males ≤ 45 years old and females ≤ 55 years old, as young adults. The THC-PAC study participants were the residents of Tehran or its suburbs who underwent coronary angiography between June 2004 and July 2011. The participants were followed via either clinical visits or phone calls at least once a year since August 2012 and had angiographically documented CAD.

This was a cross-sectional study within THC-PAC study which has been done between March 2013 and February 2014. The participants were examined at the premature CAD clinic of Tehran Heart Center and were all asked to fill in Beck Depression Inventory II. Informed consent was obtained from all participants.

***Measures***

Between March 2013 and February 2014, all patients underwent coronary angiography and CAD was defined as at least ≥ 50% luminal stenosis in individual epicardial vessels. Baseline demographics and clinical and laboratory data were collected at the time of angiography. During each clinical visit, recent clinical and laboratory data were re-collected by a trained team of physicians. 

Depressive symptoms were assessed in clinical visits using Beck Depression Inventory II, a 21-question multiple-choice self-report questionnaire. For this study, we used the Persian version of this inventory whose validity and reliability had already been assessed (9). This questionnaire is one of the most widely used instruments for measuring the presence and severity of depressive symptoms. Patients rated the severity of depressive symptoms during the previous two weeks on a four-point scale ranging from zero to three. Each of the 21 items referring to a symptom of depression is summed to give a final single score. The final sum scores ranging from zero to 13 indicate minimal or no depressive symptoms, 14 to 19 indicate mild depression, 20 to 28 indicate moderate depression and 29 to 63 indicate severe depression (10). In this study, we considered the sum score ≥ 14 as the cut-off point for defining non-depressed and depressed patients. Baseline depression status had not been yet assessed at the time of angiography. 

Body mass index (BMI) of the patients was calculated as dividing weight (in kilogram) by height (in square meters). Based on American Heart Association definition, BMI values less than 18.5 kg/m² were considered as underweight, from 18.5 kg/m² to 24.9 kg/m² as normal, from 25.0 to 29.9 kg/m² as overweight and ≥ 30.0 kg/m² as obese (11).

Major adverse cardiac events (MACE) was defined as new myocardial infarction (MI) [as the symptoms of cardiac ischemia associated with either ST elevation on the electrocardiogram (≥ 0.2 mV in leads V1, V2 and V3 and ≥ 0.1 mV in the other leads) or without ST elevation accompany by a rise in cardiac enzymes]; new coronary artery involvement (at least a new ≥ 50% luminal stenosis in an epicardial vessel besides previously detected lesions, confirmed by coronary angiography); stroke (confirmed by a neurologist); undergoing percutaneous coronary intervention (PCI); or undergoing coronary artery bypass surgery (CABG) after the initial diagnosis of CAD. Neither PCIs nor CABGs, which were done after the early stages of first diagnosis of CAD (as the early treatment strategy), were considered as MACE.

***Statistical Analysis***

Categorical variables were expressed as frequency with percentage and were compared between depressed and non-depressed groups or between the sexes using chi-squared test. Continuous variables were presented through mean and standard deviation (SD) and were compared between the sexes and also between the depressed and non-depressed groups, using Student’s t test. Backward logistic regression model, with probabilities of removal and entry as 0.1 and 0.05, respectively, was used to determine the multiple associated factors with depressive symptoms in all patients and in each sex group. All variables with p-values less than 0.2 in univariate analyses were candidates to enter the multivariable model. Effects of covariates on depressive symptoms were reported through odds ratio (OR) with 95% confidence interval (CI). Model calibration was assessed through Hosmer & Lemeshow goodness of fit test. Model discrimination power was evaluated using c-statistic which is equal to the area under the Receiver Operating Characteristic (ROC) curve. All statistical analyses were done using IBM SPSS for windows version 22 (Armonk, NY: IBM Corp).

## Results

During the clinical visit, 770 premature CAD patients, 341 men (47.7%) and 347 women (52.3%), with the mean age ± SD of 45.34±5.75 years were visited. The mean ±SD duration of follow-up was 60.43±44.32 months. The characteristics of these patients have been depicted in Table 1.

According to Beck Depression Inventory II results, 300 (39.0%) patients had mild to severe depressive symptoms. To determine which factors were associated with depressive symptoms in our premature CAD patients, we conducted univariable analysis, whose results have been depicted in Table 2.

Depressed patients were older than the non-depressed (46.14±5.75 vs.44.83±5.70 y, p = 0.002) (Table 2). Of the depressed patients, 189 (63.0%) were female, while 214 (45.5%) were female in the non-depressed group (p < 0.001). Concerning conventional risk factors, the only clearly different factor was the positive family history, which was more frequent in the depressed (p = 0.040). BMI was higher in the depressed patients (30.11±5.22 vs.28.66±4.47 kg/m2, p < 0.001). With respect to BMI categories, patients in the depressed group were more frequently obese (45.3%) while overweight was the most frequent category in the non-depressed (46.2%). MACE was significantly different between these groups and it was more commonly seen in depressed patients (24.0% vs. 17.0%, p = 0.018).

To determine the possible associated factors with depressive symptoms in premature CAD patients, we used a multivariable model; the results of this model are demonstrated in Table 3. Sex, cigarette smoking, MACE and positive family history were associated with depressive symptoms. 

Since sex was a clear determinant of depressive symptoms (OR: 2.57, 95%CI: 1.73-3.82), we stratified the subsequent analysis by sex.

Table 4 demonstrates the characteristics of the male premature CAD patients based on their depressive symptoms status; among our 367 male patients, 111 (30.2%) were depressed. The mean age of the depressed and non-depressed men were similar (41.10±3.55 vs. 41.15±3.26 y, p = 0.897). Positive family history, cigarette smoking and opium usage were significantly more frequent in depressed men (p = 0.013, p = 0.009 and p = 0.004, respectively) and hypertension was more prevalent in non-depressed men (p = 0.031). While the number of the involved vessels, EF and serum creatinine level, as indicators of the severity of the disease, were not different, MACE was more prevalent in the depressed than the non-depressed men (24.3% vs. 15.2%, p = 0.037).

aAs depicted in Table 5, in the multivariable model, opium usage had the highest odds ratio for association with depressive symptoms in male premature CAD patients (OR: 2.4), followed by MACE (OR: 2.15), initial CABG treatment (OR: 2.08), positive family history (OR: 1.83) and cigarette smoking (OR: 1.70). Hypertension found to be a protective factor (OR: 0.513).

In the 403 female patients, 189 (46.9%) were depressed. As depicted in Table 6, again, the mean age was similar in the depressed and non-depressed patients (49.11±4.62 vs. 49.24±4.81 y, p = 0.779). With respect to conventional risk factors, only hypertension showed a significant difference and it was more prevalent in the depressed than non-depressed females (69.3% vs. 59.3%, p = 0.037). The BMI of the depressed females was significantly higher than the non-depressed (31.07±5.63 vs. 29.53±4.86, 0.003). The number of involved coronary vessels, EF, serum creatinine level, abdominal circumferences, initial treatment and MACE was not significantly different between the depressed and the non-depressed females.

The results of the multivariable model for detecting possible associated factors with depressive symptoms in female premature CAD patients are presented in Table 7. In this group of patients, only hypertension (OR: 1.46) and BMI (OR: 1.06) were associated with depressive symptoms.

## Discussion

In a follow-up study of 770 patients with premature coronary artery disease, we found that the most important associated factors with depressive symptoms in men were opium usage, MACE, initial CABG treatment, positive family history for CAD and cigarette smoking. In female patients, hypertension and high BMI were associated with depressive symptoms. The prognosis of premature CAD is poorer than CAD in old age (2) and it has also been shown that depressed CAD patients have a poorer prognosis than non-depressed CAD patients (7).

Therefore, it is likely that young adults with concomitant CAD and depressive symptoms suffer from increased comorbidity and mortality. Identifying possible associated factors that could predict depressive symptoms in young adults may help the cardiologists to either control those factors or to refer at risk patients to psychiatrists.

**Table1:** Baseline Characteristics of the Recruited Patients in the Study*

	**All(n = 770)**	**Men (n = 367)**	**Women (n = 403)**	**P Value**
Age (y)	45.34±5.75	41.13±3.34	49.18±4.71	< 0.001
Risk Factors				
Positive Family History	347 (45.1)	156 (42.5)	191 (47.4)	0.173
Hypertension	366 (47.5)	108 (29.4)	258 (64.0)	< 0.001
Hyperlipidemia	555 (72.1)	255 (69.5)	300 (74.4)	0.125
Diabetes Mellitus	257 (33.4)	60 (16.3)	197 (48.9)	< 0.001
Cigarette Smoking	265 (34.4)	238 (64.9)	27 (6.7)	< 0.001
Opium Usage	77 (10.0)	75 (20.4)	2 (0.5)	< 0.001
No Risk Factor	29 (3.8)	17 (4.6)	12 (3.0)	0.228
BMI (kg/m^2^)	29.23±4.82	28.11±3.97	30.25±5.29	< 0.001
BMI Categories				< 0.001
Underweight	2 (0.3)	1(0.3)	1 (0.2)	
Normal	133 (17.3)	76 (20.7)	57 (14.1)	
Overweight	338 (43.9)	182 (49.6)	156 (38.7)	
Obese	297 (38.6)	108 (29.4)	189 (46.9)	
Creatinine(mg/dl)	0.94±0.26	1.03±0.21	0.86±0.21	< 0.001
EF (%)	52.87±9.92	51.15±10.02	54.41±9.58	< 0.001
Abdominal Circumference (cm)	98.94±11.77	98.13±9.36	99.69±13.60	0.068
Recent MI	342 (44.4)	225 (61.3)	117 (29.0)	< 0.001
No. of Involved Vessels				0.001
SVD	356 (46.2)	146 (39.8)	210 (52.1)	
2VD	208 (27.0)	105 (28.6)	103 (25.6)	
3VD	206 (26.8)	116 (31.6)	90 (22.3)	
LeftMain CoronaryInvolvement	11 (1.4)	4 (1.1)	7 (1.7)	0.450
Initial Treatment				0.245
Medical Treatment	238 (30.9)	103 (28.1)	135 (33.5)	
PCI	349 (45.3)	171 (46.6)	178 (44.2)	
CABG	183 (23.8)	93 (25.3)	90 (22.3)	
MACE	152 (19.7)	66 (18.0)	86 (21.3)	0.243
Duration of F/U (mo)	60.43±44.32	58.96±20.92	61.77±57.94	0.381

BMI, Body mass index; EF, Ejection fraction; MI, Myocardial infarction; SVD, Single-vessel disease; 2VD, Two-vessel disease; 3VD, Three-vessel disease; PCI, Percutaneous coronary intervention; CABG, Coronary artery bypass grafting; MACE; Major adverse cardiac events; F/U, Follow-up

* Data are presented as mean± SD or n (%)

**Table2:** Determinants of Depressive Symptoms in the Recruited Patients of the Study (n = 770)*

	**Non-Depressed (n = 470)**	**Depressed (n=300)**	**P Value**
Age (y)	44.83±5.70	46.14±5.75	0.002
Sex			< 0.001
Male	256 (54.5)	111 (37.0)	
Female	214 (45.5)	189 (63.0)	
Risk Factors			
Positive Family History	198 (42.1)	149 (49.7)	0.040
Hypertension	211 (44.9)	155 (51.7)	0.066
Hyperlipidemia	340 (72.3)	215 (71.7)	0.839
Diabetes Mellitus	146 (31.3)	111 (37.0)	0.088
Cigarette Smoking	171 (36.4)	94 (31.3)	0.150
Opium Usage	42 (8.9)	35 (11.7)	0.218
No Risk Factor	18 (3.8)	11 (3.7)	0.908
BMI (kg/m2)	28.66±4.47	30.11±5.22	< 0.001
BMI Categories			0.015
Underweight	1 (0.2)	1 (0.3)	
Normal	91 (19.4)	42 (14.0)	
Overweight	217 (46.2)	121 (40.3)	
Obese	161 (34.3)	136 (45.3)	
Creatinine(mg/dl)	0.95±0.22	0.92±0.23	0.072
EF (%)	52.90±10.00	52.81±9.81	0.912
Abdominal Circumference (cm)	98.32±11.48	99.90±12.16	0.074
Recent MI	215 (45.7)	127 (42.3)	0.353
No of Involved Vessels			0.379
SVD	211 (44.9)	145 (48.3)	
2VD	125 (26.6)	83 (27.7)	
3VD	134 (28.5)	72 (24.0)	
Left Main Coronary Involvement	7 (1.5)	4 (1.3)	0.859
Initial Treatment			0.776
Medical Treatment	145 (30.9)	93 (31.0)	
PCI	217 (46.2)	132 (44.0)	
CABG	108 (23.0)	75 (25.0)	
MACE	80 (17.0)	72 (24.0)	0.018
Duration of F/U (mo)	59.34±22.24	62.14±65.37	0.394

BMI, Body mass index; EF, Ejection fraction; MI, Myocardial infarction; SVD, Single-vessel disease; 2VD, Two-vessel disease; 3VD, Three-vessel disease; PCI, Percutaneous coronary intervention; CABG, Coronary artery bypass grafting; MACE, Major cardiac adverse events; F/U, Follow-up

* Data are presented as mean± SD or n (%)

**Table3:** Multiple Associated Factors with Depressive Symptoms in all Patients with Premature CAD

	**Wald Statistic**	**P Value**	**Odds Ratio**	**95% Confidence Interval**	
				**Lower**	**Upper**
Positive Family History	2.912	0.088	1.299	0.962	1.753
MACE	5.080	0.024	1.526	1.057	2.203
Cigarette Smoking	3.935	0.047	1.626	1.005	2.317
Female Sex	21.592	< 0.001	2.567	1.725	3.820

Hosmer-Lemeshow test, P = 0.201; the area under the ROC curve: 0.621 (95% CI: 0.581, 0.661), p<0.001

CAD, Coronary artery disease; MACE, Major adverse cardiac events

**Table4:** Demographic, Clinical and Paraclinical Comparison between Male Patients with and Without Depressive Symptoms (n = 367)*

	**Non-Depressed Male (n=256)**	**Depressed Male (n=111)**	**P Value**
Age (y)	41.15±3.26	41.10±3.55	0.897
Risk Factors			
Positive Family History	98 (38.3)	58 (52.3)	0.013
Hypertension	84 (32.8)	24 (21.6)	0.031
Hyperlipidemia	184 (71.9)	71 (64.0)	0.131
Diabetes Mellitus	41 (16.0)	19 (17.1)	0.793
Cigarette Smoking	155 (60.5)	83 (74.8)	0.009
Opium Usage	42 (16.4)	33 (29.7)	0.004
No Risk Factor	13 (5.1)	4 (3.6)	0.537
BMI (kg/m2)	27.95±3.98	28.50±3.95	0.227
BMI Categories			0.586
Underweight	1 (0.4)	0	
Normal	57 (22.3)	19 (17.1)	
Overweight	126 (49.2)	56 (50.5)	
Obese	72 (28.1)	36 (32.4)	
Creatinine (mg/dl)	1.03±0.20	1.04±0.22	0.649
EF (%)	51.65±10.02	49.97±9.96	0.156
Abdominal Circumference (cm)	98.02±9.31	98.39±9.53	0.726
Heart Failure	1 (0.4)	0	1.0
No of Involved Vessels			0.817
SVD	102 (39.8)	44 (39.6)	
2VD	71 (27.7)	34 (30.6)	
3VD	83 (32.4)	33 (29.7)	
Left Main Coronary Involvement	2 (0.8)	2 (1.8)	0.387
Initial Treatment			0.101
Medical Treatment	77 (30.1)	26 (23.4)	
PCI	122 (47.7)	49 (44.1)	
CABG	57 (22.3)	36 (32.4)	
CAD Duration (mo)	58.13±20.50	58.23±21.02	0.968
MACE	39 (15.2)	27 (24.3)	0.037

BMI, Body mass index; EF, Ejection fraction; MI, Myocardial infarction; SVD, Single-vessel disease; 2VD, Two-vessel disease; 3VD, Three-vessel disease; PCI, Percutaneous coronary intervention; CABG, Coronary artery bypass grafting; CAD, Coronary artery disease; MACE, Major cardiac adverse events

* Data are presented as mean ±SD or n (%)

**Table5:** Multiple Associated Factors with Depressive Symptoms in Male Patients with CAD

	**Wald Statistic**	**P Value**	**Odds Ratio**	**95% Confidence Interval**	
				**Lower**	**Upper**
Positive Family History	5.542	0.019	1.825	1.106	3.011
Hypertension	5.074	0.024	0.513	0.287	0.917
Cigarette Smoking	3.434	0.064	1.699	0.970	2.977
Opium Usage	8.254	0.004	2.423	1.325	4.433
Medical Treatment	5.300	0.071			
PCI	0.349	0.555	1.205	0.649	2.240
CABG	4.626	0.031	2.082	1.067	4.062
MACE	6.131	0.013	2.146	1.173	3.928

Hosmer-Lemeshow test, P = 0.117; the area under the ROC curve: 0.689 (95% CI: 0.631, 0.746), p<0.001

CAD, Coronary artery disease; PCI, Percutaneous coronary intervention; CABG, Coronary artery bypass grafting; MACE, Major adverse cardiac events

**Table6:** Demographic, Clinical and Paraclinical Comparison between Female Patients with and Without Depressive Symptoms (n = 403)*

	**Non-Depressed Female (n=214)**	**Depressed Female (n=189)**	**P Value**
Age (y)	49.24±4.81	49.11±4.62	0.779
Risk Factors			
Positive Family History	100 (46.7)	91 (48.1)	0.776
Hypertension	127 (59.3)	131 (69.3)	0.037
Hyperlipidemia	156 (72.9)	144 (76.2)	0.449
Diabetes Mellitus	105 (49.1)	92 (48.7)	0.938
Cigarette Smoking	16 (7.5)	11 (5.8)	0.507
Opium Usage	0	2 (1.1)	0.219
No Risk Factor	5 (2.3)	7 (3.7)	0.420
BMI (kg/m^2^)	29.53±4.86	31.07±5.63	0.003
BMI Categories			0.087
Underweight	0	1 (0.5)	
Normal	34 (15.9)	23 (12.2)	
Overweight	91 (42.5)	65 (34.4)	
Obese	89 (41.6)	100 (52.9)	
Creatinine (mg/dl)	0.87±0.22	0.86±0.21	0.632
EF (%)	54.41±9.79	54.42±9.38	0.997
Abdominal Circumference (cm)	98.69±13.70	100.80±13.44	0.129
Recent MI	57 (26.6)	60 (31.7)	0.259
No of Involved Vessels			0.741
SVD	109 (50.9)	101 (53.4)	
2VD	54 (25.2)	49 (25.9)	
3VD	51 (23.8)	39 (20.6)	
Left Main Coronary Involvement	5 (2.3)	2 (1.1)	0.327
Initial Treatment			0.648
Medical Treatment	68 (31.8)	67 (35.4)	
PCI	95 (44.4)	83 (43.9)	
CABG	51 (23.8)	39 (20.6)	
CAD Duration (mo)	59.02±23.57	57.39±21.58	0.472
MACE	41 (19.2)	45 (23.8)	0.255

BMI, Body mass index; EF, Ejection fraction; MI, Myocardial infarction; SVD, Single-vessel disease; 2VD, Two-vessel disease; 3VD, Three-vessel disease; PCI, Percutaneous coronary intervention; CABG, Coronary artery bypass grafting; CAD, Coronary artery disease; MACE, Major cardiac adverse events

* Data are presented as mean ±SD or n (%)

**Table7:** Multiple Associated Factors with Depressive Symptoms in Female Patients with CAD

	Wald Statistic	P Value	Odds Ratio	95% Confidence Interval	
				Lower	Upper
Hypertension	3.170	0.075	1.462	0.962	2.220
BMI	7.293	0.007	1.055	1.015	1.097

Hosmer-Lemeshowtest, P=0.863; the area under the ROC curve: 0.598 (95% CI: 0.543, 0.653), p = 0.001

CAD, Coronary artery disease; BMI, Body mass index

 This may improve the prognosis for depressed premature CAD patients and increase life expectancy and quality of life. 

In our study, 20.4% of the male patients were opium users and opium had the greatest association with depressive symptoms in this group of patients, with an over two-fold increase in risk. With respect to collecting data on the opium usage of the patients, we only considered the self-reporting response of the patients at the time of data collection.

Therefore, we would rather use the term “opium usage” rather than “opium abuse” or “opium dependence”. 

Opium is among the most used substances in the Middle East and Iran, with a history dating back thousands of years. A previous study in opium addicted individuals showed that depressive symptoms can be considered as one of the clinical symptoms of drug addiction (12). Gharagozlou et al. have indicated that in most Western studies on drug addicts, mainly including addiction to other substances, anxiety and evidence of antisocial behavior were reported as the most common findings, while the most frequent symptom among opium addicts was depression (13). Our results are in line with these findings although anxiety was highly prevalent.

Mace (MI, new coronary involvement, stroke, or revascularization) was the other associated factor with depressive symptoms in the male young adults with CAD (OR: 2.15, 95% CI: 1.17-3.93). In our patients, MACE was more prevalent in depressed patients than the non-depressed. Frasure-Smith et al. assessed the 2-year cardiac prognostic importance of depression in 804 patients with stable CAD. They found that MACE (cardiac death, MI, cardiac arrest, or non-selective revascularization) was 30% more frequent in depressed CAD patients than in the non-depressed (14). In another study by Frasure-Smith et al. conducted on patients who developed immediate post MI depression, 4-6 times increased risk of cardiac mortality was found between 6 to 18 months after MI (15). Barefoot et al. followed 1,250 patients with CAD up to 19.4 years and they showed that depressed patients had 69% higher risk for cardiac death (16). Depression not only may increase the risk of MACE in CAD patients, but also non-CAD patients with depression are more likely to die of cardiac causes. In a prospective study on 2,397 patients who were free of CAD, Penninx and her colleagues found that depressed patients had 3.9-fold higher risk of dying of a cardiac cause than those without depression even after controlling for risk factors and severity of the disease (7). Even though several studies have reported increased risk of cardiac events in depressed patients, there are some studies with null findings, small effect size, or inconsistency across certain subgroups, most of which suffer from small sample size, limited follow-ups, or inadequate depression assessment (5, 17). 

Initial CABG treatment was one of the associated factors with depressive symptoms in the premature CAD patients, doubling the risk. The point prevalence of depressive symptoms in the young CABG patients was 41.0%, while this prevalence has been reported from 20% to 30% in all-age CABG patients in other studies (18-20). Depressive symptoms are not only common in CABG patients, but they also increase the risk of cardiac events in these patients. A study by Connerney et al. on CABG patients showed that depressed patients were more than twice as likely to have cardiac events within 12 months after surgery (19). In addition, Blumenthal et al. found that depression was associated with a two to three times increased death incidence in CABG patients (18). 

Our study revealed that positive family history of CAD is also associated with depressive symptoms. Depressive symptoms were present in 42.9% of the depressed and 35.7% of the non-depressed patients. Association of depressive symptoms and positive family history of CAD may implicate the role of genetic factors for both depressive symptoms and CAD, which needs to be further investigated in the future. Furthermore, it is highly likely that individuals with positive family history of CAD to have family members with cardiac problems, or they even may be afraid of developing CAD in the near future, which could make them depressed. These points may be considered as the other plausible causes for this association.

Cigarette smoking was the other associated factor with depressive symptoms in the male patients, with a 70% increased risk. A recent meta-analysis has examined 85 relevant studies to determine the association of smoking and depressive symptoms (21). The results of this analysis revealed that current smokers were more likely to be depressed than never smokers were (OR = 1.50, CI = 1.39-1.60) and that depressive symptoms were more prevalent in current than former smokers were (OR = 1.76, CI = 1.48-2.09). Moreover, it was found that smoking cessation could improve depressive symptoms (22).

In this study, hypertension showed a protective role in men, reducing the risk of depressive symptoms by half and a risk factor in women, with a 50% increased risk. Female patients were more often depressed (46.9% vs. 30.2%, p < 0.001) and were more likely to be hypertensive (64.8% vs. 28.4%, p < 0.001) than men. Some mechanisms may underlie sex differences in the relations between depressive symptoms and hypertension. Depression is associated with hypothalamic-pituitary-adrenal (HPA) axis dysregulation which may increase the risk for hypertension (23). Women are more likely than males to experience depression in response to stressors and certain stressful events; for instance, work–family tensions are more prevalent in women, which may chronically activate the HPA axis (24). Furthermore, depression has a well-known association with inflammatory factors (25), which may play a role in the development of hypertension. It has been shown that inflammatory processes are more likely to influence CAD development in women than in men (26). For instance, the blood levels of C-reactive protein are higher in women than in men at all ages (26). Additionally, there are some bio behavioral risk factors such as physical inactivity, obesity and sleep disorders that are associated with both depression and hypertension, which are more prevalent in women than in men (24).

BMI was the other associated factor with depressive symptoms in the female patients. Same as depressive symptoms, obesity was also more prevalent in our female patients (45.0% vs. 26.6%, p < 0.001). Increased BMI is associated with several chronic diseases, most of which are associated with psychiatric disorders (27). Zhao et al. analyzed the data collected from 177,047 participants and they found that depressive symptoms were more frequently seen in women who were overweight or obese (27). However, there are some studies, which show an inverse relationship between BMI > 30 kg/m2 and depression, as they were less depressed than those with lower BMI. They showed a difference in the frequency of depression in people with different BMI levels independent of the disease status or other psychosocial or lifestyle factors. Moreover, a recent study on more than 6,000 participants in Puerto Rico has shown that depression is associated with obesity, particularly in women with severe obesity, less education and lower incomes (28).

## Limitations

This study suffers from an important limitation, which is the absence of baseline depressive symptoms status. Hence, it remains unknown how many of the depressed patients had already been depressed at the time of CAD diagnosis and how many of them developed depressive symptoms thereafter. This hampers the firm establishment of temporal and causal associations. Furthermore, no information was available on the history of depressive disorders in patients’ family members. Another limitation of this study was the absence of the knowledge about the psychiatric drugs the patients were using at the time of data collection. However, strength of this study was the inclusion of a large proportion of women, contrary to many previous studies. Our results revealed the relevance of studying both sexes since determinants of depressive symptoms appear to differ.

## Conclusion

In this study, it was found that opium usage, MACE, initial CABG treatment, positive family history for CAD and cigarette smoking are associated factors with depressive symptoms in young male patients with documented CAD. However, these factors were different in women and hypertension and BMI were associated with depressive symptoms in the female patients. These findings may have important clinical consequences.
